# The long multi-epitope peptide vaccine combined with adjuvants improved the therapeutic effects in a glioblastoma mouse model

**DOI:** 10.3389/fimmu.2022.1007285

**Published:** 2022-11-09

**Authors:** Thi-Anh-Thuy Tran, Young-Hee Kim, Ga-Eun Kim, Shin Jung, In-Young Kim, Kyung-Sub Moon, Young-Jin Kim, Tae-Kyu Lee, Hyosuk Yun, Je-Jung Lee, Hyun-Ju Lee, Chul Won Lee, Tae-Young Jung

**Affiliations:** ^1^ Brain Tumor Research Laboratory, Chonnam National University Hwasun Hospital, Hwasun, South Korea; ^2^ Biomedical Sciences Graduate Program (BMSGP), Chonnam National University Medical School, Hwasun, South Korea; ^3^ Chonnam National University Medical School, Chonnam National University, Hwasun, South Korea; ^4^ Department of Neurosurgery, Chonnam National University Medical School, and Hwasun Hospital, Hwasun, South Korea; ^5^ Department of Chemistry, Chonnam National University, Gwangju, South Korea; ^6^ Research Center for Cancer Immunotherapy, Chonnam National University Hwasun Hospital, Hwasun, South Korea; ^7^ Department of Internal Medicine, Chonnam National University Medical School, and Hwasun Hospital, Hwasun, South Korea

**Keywords:** long multi-epitope peptide, BIRC5, EphA2, lenalidomide, anti-PD1

## Abstract

Emerging data have suggested that single short peptides have limited success as a cancer vaccine; however, extending the short peptides into longer multi-epitope peptides overcame the immune tolerance and induced an immune response. Moreover, the combination of adjuvants such as lenalidomide and anti-programmed cell death protein 1 (PD1) with a peptide vaccine showed potential vaccine effects in previous studies. Therefore, the effects of a long multi-epitope peptide vaccine in combination with lenalidomide and anti-PD1 were analyzed in this study. Long multi-epitope peptides from two MHCI peptides (BIRC5^97-104^ and EphA2^682-689^) and the pan-human leukocyte antigen-DR isotype (HLA-DR) binding epitope (PADRE) were synthesized. The therapeutic effects of long multi-epitope peptides in combination with lenalidomide and anti-PD1 were confirmed in the murine GL261 intracranial glioma model. Immune cells’ distribution and responses to the long multi-epitope peptides in combination with these adjuvants were also estimated in the spleens, lymph nodes, and tumor tissues. The difference between long multi-epitope peptides and a cocktail of multi-epitope peptides combined with lenalidomide and anti-PD1 was also clarified. As a result, long multi-epitope peptides combined with lenalidomide and anti-PD1 prolonged the survival of mice according to the suppression of tumor growth in an intracranial mouse model. While long multi-epitope peptides combined with these adjuvants enhanced the percentages of activated and memory effector CD8^+^ T cells, the increase in percentages of regulatory T cells (Tregs) was observed in a cocktail of multi-epitope peptides combined with lenalidomide and anti-PD1 group in the tumors. Long multi-epitope peptides combined with these adjuvants also enhanced the function of immune cells according to the enhanced pro-inflammatory cytokines and cytotoxicity against GL261 cells in *ex vivo*. In conclusion, long multi-epitope peptides composed of MHCI peptides, BIRC5 and EphA2, and the MHCII peptide, PADRE, in combination with lenalidomide and anti-PD1 has the potential to improve the therapeutic effects of a vaccine against GBM.

## Introduction

The immune system has shown a role in controlling tumors, and immunotherapy has revolutionized cancer treatment; however, the efficacy remains limited in most clinical settings, especially in glioblastoma (GBM) (1, 2). While numerous peptides from tumor-associated antigens (TAAs) or neoantigens (TSAs) were identified to augment tumor-specific T cell responses in GBM, the therapeutic effects were insufficient in impacting the survival of GBM patients. Targeting multiple antigens in the development of peptide vaccines were investigated and resulted in improved effective cytotoxic T lymphocyte (CTL) response and managed to avoid tumor immune escape due to the heterogeneity characteristics (3, 4). Vaccination strategies utilizing individual CD8^+^ T cell epitopes alone do not routinely produce significant clinical responses. It is critical to stimulate CD4^+^ helper T cells to enhance CD8^+^ CTL responses through direct and indirect mechanisms (5). Using the universal pan-human leukocyte antigen-DR isotype (HLA-DR) binding epitope (PADRE) can improve therapeutic responses to major histocompatibility complex (MHC) I-restricted epitopes by enhancing the production of interferon (IFN)-γ (6, 7).

Short peptides have displayed low MHC-binding affinity and fail to elicit a robust CTL response. However, extending the short peptides into longer peptides can overcome immune tolerance and induce T cell responses. After vaccination, short peptides directly bind to MHCI molecules expressed by all nucleated cells, most of which are not specialized for antigen presentation; thus, there is suboptimal T-cell priming and tolerance. However, long peptides must be taken up and processed by professional antigen-presenting cells (APCs) for presentation and T cell activation, thus alleviating the potential immune tolerance and enhancing the vaccine potency (7, 8). Therefore, a long multi-epitope peptide vaccine was designed using two common TAA antigen peptides in GBM (BIRC5 and EphA2), and the universal PADRE with the purpose of stimulating CD4^+^ and CD8^+^ T cells was investigated in this study.

Although lenalidomide has been shown to improve progression-free survival (PFS) and overall survival (OS) in multiple myeloma (MM) (9), the effects of lenalidomide alone have not been shown in GBM. Lenalidomide had little effect on the induction of apoptosis in GBM cells; however, it regulated the functions of CAR T cells and improved patient outcomes in combination with peptide vaccines in previous studies (10–12). The combination of the peptide vaccine and an anti-programmed cell death protein 1 (PD1) antibody therapy worked synergistically against GBM in a previous study (13). Therefore, this study aims to investigate the therapeutic potential of a vaccine using long multi-epitope peptides composed of MHCI (BIRC5 and EphA2) and MHCII peptides (PADRE) in combination with lenalidomide and anti-PD1 to enhance CTLs function against GBM in a mouse model.

## Materials and methods

### Animals and cell lines

Six- to eight-week-old female C57BL/6 mice (H-2b, I-Ab) were purchased from Orient Bio (Iksan, Republic of Korea). Mice were raised under specific pathogen-free (SPF) conditions. The mice were anesthetized by intraperitoneal injection (i.p) of a 2:1 mixture of Zoletil^®^ (Virbac Laboratories, Corros, France)/Rompun^®^ (Bayer Korea, Anshan, Korea) at a dose of 1.5 mL/kg.

Animal research was carried out in compliance with the Animal Research: Reporting of *In Vivo* Experiments (ARRIVE) guidelines (14). Mice that did not develop tumors or died during the experiments were excluded from this study. Cells from mice that were not in good conditions, such as having too many dead cells or a lot of cell debris, due to experimental techniques or samples that did not have enough cell numbers to perform the experiments were excluded. Moreover, cells from mice missed during the sample loading in ELISA, ELISPOT, and LDH assay due to experimental techniques were also excluded. All animal care, experiments, and euthanasia were performed under the approval of the Chonnam National University Animal Research Committee.

The murine glioblastoma cell line, GL261: H-2b and I-Ab (provided by Dr. Maciej S. Lesniak, Northwestern University) and the murine lymphoma cell line, YAC-1 (American Type Culture Collection [ATCC], Rockville, MD, USA) were used for cell culture. GL261 cells were maintained in Dulbecco’s Modified Eagle Medium (DMEM), and YAC-1 cells were grown in Roswell Park Memorial Institute (RPMI) 1640 medium supplemented with 10% fetal bovine serum (FBS) and 1% penicillin-streptomycin (P/S) at 37°C in 5% CO_2_.

### Peptide synthesis and antibodies

All single peptides composed of the murine BIRC5 peptide (H-2b-restricted BIRC5^97-104^: TVSEFLKL), the murine EphA2 peptide (H-2b-restricted EphA2^682–689^: VVSKYKPM), and the PADRE peptide (I-Ab-restricted PADRE, ak-Cha-VAAWTLKAAa-Z-C) were commercially synthesized by the Peptron Company (Daejeon, Korea) with a purity greater than 95% by reverse-phase high-performance liquid chromatography (HPLC). The long multi-epitope peptides were synthesized in the laboratory of Prof. Chul Won Lee at the Chemistry Department (Chonnam National University). The long multi-epitope peptides were generally made by incorporating two single peptides (BIRC5^97-104^ and EphA2^682–689^) with a PADRE. The binding scores of BIRC5 and EphA2 peptides were predicted using SYFPEITHI: http://www.syfpeithi.de. All peptides were dissolved in dimethyl sulfoxide (DMSO) and diluted with phosphate-buffered saline (PBS). Mouse anti-PD1 (clone RMP1-14) used for the *in vivo* blockade was purchased from BioXcell (West Lebanon, NH, USA).

### The 3-(4,5-dimethylthiazol-2-yl)-2,5-diphenyltetrazolium bromide (MTT) cell viability assay

The effects of lenalidomide on the proliferation of the GL261 cell line were estimated by the 3-(4,5-dimethylthiazol-2-yl)-2,5-diphenyltetrazolium bromide (MTT) cell viability assay. Briefly, GL261 cells (5 x 10^3^ cells/well) were treated with lenalidomide according to different doses (2.5, 5, 10, 20 µg/mL) and seeded in 96-well plates cultured with DMEM supplemented with 10% FBS and 1% P/S at 37°C, 5% CO_2_. After that, the cells were stained every 24 hours until day five with MTT (Sigma, USA). For staining, the plates were washed with PBS, and MTT (0.5 mg/mL) was added to each well. The MTT solution was removed from each well after four hours of incubation. MTT formazan was solubilized in Isopropanol (Merck, Germany), and the optical density was read at 570 nm.

### Intracranial glioma mouse model and treatment schedule

To establish the mouse intracranial model, 1 × 10^5^ GL261 cells in 5 µL of PBS were stereotactically injected into the right striatum at a rate of 1 µL/min. Injection sites were estimated by the following coordinates: 1 mm posterior, 2 mm lateral from bregma, and 4 mm deep from the cortical surface (15). Mice were randomly allocated into the treatment arm. Mice were divided into the following six treatment groups: 1) no treatment; 2) long multi-epitope peptide vaccine; 3) long multi-epitope peptide vaccine plus lenalidomide; 4) lenalidomide plus anti-PD1; 5) long multi-epitope peptide vaccine plus lenalidomide and anti-PD1; 6) a cocktail of multi-epitope peptide vaccine plus lenalidomide and anti-PD1. On day one and day six post-tumor inoculation, the mice were intraperitoneally injected with lenalidomide (0.5 mg/injection). After that, long multi-epitope peptides or a cocktail of multi-epitope peptides (300 µg/injection) were intramuscularly administrated on days 2, 7, and 12. Mice were also administered with intraperitoneal injections of MAb anti-mouse PD1 (200 µg/injection) every three days (days 5, 8, 11, and 14). The survival was quantified. Mice were also euthanized on day 20 to assess the immunological parameters in the spleens, lymph nodes, and tumors.

### Splenocytes, lymph nodes, and single-tumor cell isolation

Splenocytes, lymph nodes, and single-tumor cells were isolated directly from the spleens, lymph nodes, and tumors of nonvaccinated and vaccinated mice. For splenocyte isolation, spleens were collected and washed with RPMI media supplemented with 10% FBS and 1% P/S. A 1 mL syringe plunger was used to gently press the spleen through the 100 µm cell strainer (Falcon, USA) while continuously adding media. After filtering with a 40 µm cell strainer (Falcon, USA), the cells were collected and washed with media. For single-tumor cell and lymph nodes isolation, the tumors and lymph nodes were collected and washed with RPMI media supplemented with 10% FBS and 1% P/S. Afterward, the tumors were minced into 3–4 mm pieces with a sterile scalpel. Tumor pieces and lymph nodes were incubated with collagenase type IV (0.25%; Gibco, USA) at 37°C, 5% CO_2_ for two hours. Samples were observed and suspended at 15-minute intervals. Cells were filtered with 100 µm and 40 µm cell strainers (Falcon, USA), and single-tumor cells or lymph nodes were collected. Erythrocytes were removed from all samples using a red blood cells lysis solution (Multenyi Biotech, Bergisch Gladbach, Germany).

### Flow cytometry

For the *ex vivo* experiments, splenocytes, lymph nodes, and tumor-single cells were stained to confirm immune cells. For cell surface staining, cells were stained with anti-mouse CD45-Pacific Blue, anti-mouse CD3-PE/Cyanine7, anti-mouse CD4-PE, anti-mouse CD8-PE, anti-mouse CD44-APC, anti-mouse CD62L-FITC, anti-mouse CD25-FITC, anti-mouse CD69-FITC, anti-mouse CD49b-PE, anti-mouse CD279 (PD1)-FITC, or anti-mouse CD274 (PDL1)-PE for 30 minutes at 4°C. For intracellular staining, cells were stained with surface markers such as anti-mouse CD45-Pacific Blue, anti-mouse CD3-PE/Cyanine7, anti-mouse CD4-PE, anti-mouse CD8-PE, or anti-mouse CD25-FITC for 30 minutes at 4°C. Cells were washed and permeabilized with FACS™ Permeabilizing Solution 2 (BD Biosciences) for 30 minutes at 20–22°C. After washing twice with the permeabilization buffer, cells were stained with anti-mouse Forkhead box P3 (Foxp3)-Alexa Fluor^®^ 647 or anti-mouse IFN-γ-APC/Cyanine7 for 30 minutes at 4°C. For IFN-γ staining, the Protein Transport Inhibitor containing Brefeldin A (BD Golgi Plug™) was added at 1 µl per 1 x 10^6^ cells/well and incubated for five hours. The information about all used antibodies is listed in **Table 1**. All flow cytometry data were acquired on a BD FACs Canto II (Becton Dickinson, Mountain View, CA, USA). All data were analyzed using FlowJo v10 software (TreeStar, San Carlos, CA, USA).

**Table 1:** List of antibodies used in flow cytometry.

Name	Catalog #	Clone	Company
LIVE/DEAD^TM^ Fixable Dead Cell Stain Kits	L34966		lnvitrogen
Pacific Blue^TM^ anti-mouse CD45 Antibody	103126	30-F11	BioLegend
PE/Cyanine7 anti-mouse CD3 Antibody	100220	17A2	BioLegend
PE Rat Anti-Mouse CD4	557308	GK l.5	BD Pharmingen^TM^
PE Rat Anti-Mouse CD8a	553033	53-6.7	BD Pharmingen^TM^
PE Rat Anti-Mouse CD49b	553858	DX5	BD Pharmingen^TM^
FITC Hamster Anti-Mouse CD69	553236	H l .2F3	BD Pharmingen^TM^
APC Monoclonal Antibody CD44	l7-0441-82	IM7	lnvitrogen
Alexa Fluor® 647 anti-Mouse Foxp3	560401	MF23	BD Pharmingen^TM^
FITC Monoclonal Anti body CD279 (PD1)	11-9985-82	J43	Invitrogen
FITC Rat Anti-Mouse CD25	558689	3C7	BD Pharmingcn^TM^
FITC Rat Anti-Mouse CD62L	553150	MEL-14	BD Pharmingen^TM^
APC/Cyanine7 Anti-mouse IFNy	505850	XMG l.2	BioLegend
PE Rat Anti-Mouse CD274	558091	MIH5	BD Pharmingen^TM^

### Serum collection, splenocytes and lymph nodes re-stimulation, and single-tumor cells *ex vivo* culture

After the mice were anesthetized, blood was withdrawn slowly from the heart by performing a thoracotomy. To collect the serum, the blood was kept at room temperature for one hour without an anticoagulation treatment; the clot was removed by centrifugation at 2000 rpm for ten minutes using a refrigerated centrifuge. The resulting supernatant was designated serum, collected, and stored at -80°C for analysis.

Splenocytes and lymph nodes were restimulated according to the following protocol. Splenocytes and lymph nodes isolated from non-vaccinated and vaccinated mice after the last immunization were cultured in 24-well plates (1 × 10^6^ cells/well) and re-stimulated with the cocktail of multi-epitope peptides or long multi-epitope peptides (30 µg/mL) for four days in RPMI-1640 (Gibco-BRL) prepared in 10% FBS with 1% P/S supplements and recombinant mouse (rm) IL-2 (20 ng/mL) (R&D Systems). Anti-PD1 (10 µg/mL) was added during re-stimulation. After re-stimulation, the supernatant and cells were collected and analyzed for immune cell functions.

Single-tumor cells from the tumor were cultured in 24 well-plates (1 x 10^6^ cells/well) for 24 hours at 37°C, 5% CO_2,_ and the supernatant was collected for pro-inflammatory and anti-inflammatory cytokine determination by an enzyme-linked immunoassay (ELISA).

### IFN-γ release Enzyme-Linked ImmunoSpot assay

The re-stimulated splenocytes and re-stimulated lymph nodes were examined for IFN-γ secretion using the IFN-γ release Enzyme-Linked ImmunoSpot (ELISPOT) assay kit (BD Biosciences). Ninety-six-well PVDF membrane ELISPOT plates (Millipore, USA) were coated with the capture-purified anti-mouse IFN-ɣ antibody overnight at 4°C. Then, RPMI medium supplemented with 10% FBS was added to saturate the treated antibodies. The re-stimulated splenocytes and re-stimulated lymph nodes from immunized mice were co-cultured with the target cells (GL261 and YAC-1 cell line; 2×10^4^ cells/well) at a ratio of 1: 10 (target: effector). Co-cultured cells were incubated in a 10% FBS-RPMI medium for 24 hours at 37°C and 5% CO_2_. The plates were incubated for two hours with the biotinylated detection anti-mouse IFN-ɣ antibody and for one hour with streptavidin-HRP. After washing, spots were revealed using an AEC substrate reagent set (BD Bioscience) and measured on an automatic CTL Immunospot Analyzer (Cellular Technology Ltd., USA).

### LDH release cytotoxicity assay

A CytoTox 96 nonradioactive cytotoxicity assay (CytoTox 96, Promega, USA) was performed to analyze the killing effect of the re-stimulated splenocyte effector cells and the re-stimulated lymph nodes against target cancer cells according to the manufacturer’s instructions. GL261 and YAC-1 cell lines (2×10^4^ cells/well) were used as the target cells. The re-stimulated splenocytes and re-stimulated lymph nodes were co-cultured with the target cells at a ratio of 1: 10 (target: effector) in uncoated 96-well plates (Costar, USA) for five hours at 37°C and 5% CO2. Then, supernatants were collected for a lactate dehydrogenase concentration assay. The mean percentage of specific lysis was calculated as following: % Cytotoxicity = [(Experimental - Effector Spontaneous - Target Spontaneous)/(Target Maximum - Target Spontaneous)] × 100

### Enzyme-linked immunosorbent assay

The levels of pro-inflammatory and anti-inflammatory cytokines in the serum, culture media of the re-stimulated splenocytes, re-stimulated lymph nodes, and single-tumor cells from non-vaccinated and vaccinated mice were estimated using the OptEIA ELISA kit (BD Bioscience) according to the manufacturer’s instructions. The serum was used to analyze the pro-inflammatory cytokines (IFN-γ). Culture media from re-stimulated splenocytes and re-stimulated lymph nodes were used to estimate the change of pro-inflammatory (IL-12p70 and IFN-γ) and anti-inflammatory cytokines (IL-10). The culture media of single-tumor cells was harvested for the quantitation of pro-inflammatory (IFN-γ) and anti-inflammatory (transforming growth factor-beta [TGF-β] and IL-10) cytokines.

### Statistical analysis

All statistical analyses were performed using SPSS 23.0 for Windows (SPSS Inc., Chicago, IL, USA). Two-way and one-way analysis of variance (ANOVA) was performed across the multiple groups. A log-rank test was performed on the survival data. A p-value of less than 0.05 was considered statistically significant. In the experiment, n refers to the number of animals, and the individual mouse was considered the experimental unit within the studies. Each experiment was done individually with a minimum of two mice per group and repeated three times. The mouse number used for each experiment was described in the figure legends of each experiment. All data was displayed as the mean ± standard deviation (*SD*) (excepting the data for mouse tumor volume, which was shown as the mean ± standard error of the mean [*SEM*]).

## Results

### Therapeutic effects of a long multi-epitope peptide vaccine combined with lenalidomide and anti-PD1 on the GBM mouse model

**Figure 1:**
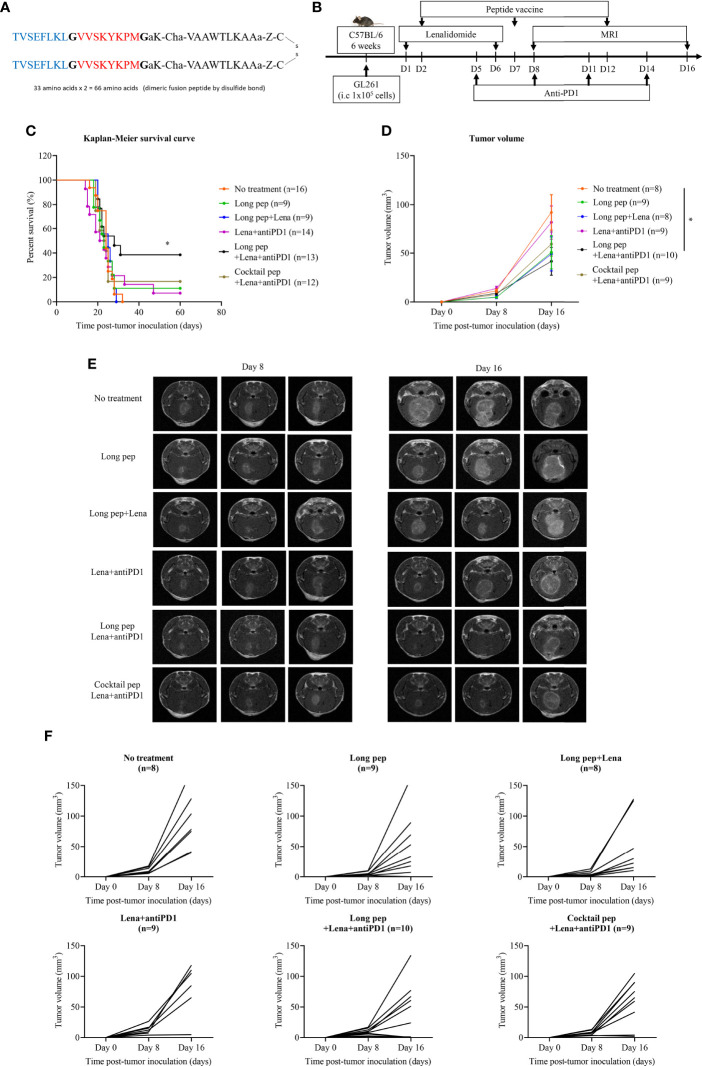
The construction of the long multi-epitope peptide vaccine **(A)**. Schema outlining treatments used in this experiment **(B)**. The therapeutic effects of the peptide vaccine combined with lenalidomide and anti-PD1 was clarified. Kaplan-Meier survival curves were used to determine the survival of the mouse tumor model according to the different combination treatments in GL261 glioma-bearing mice (No treatment: n = 16, long pep: n = 9, long pep+Lena: n = 9, Lena+anti-PD1: n = 14, long pep+Lena+anti-PD1: n = 13, and cocktail pep+Lena+anti-PD1: n = 12) **(C)**. Brain tumor sizes after treatment with the different combinations were confirmed using magnetic resonance imaging (MRI) (No treatment: n = 8, long pep: n = 9, long pep+Lena: n = 8, Lena+anti-PD1: n = 9, long pep+Lena+anti-PD1: n = 10, and cocktail pep+Lena+anti-PD1: n = 9). The tumor volume was calculated by summating all tumor areas in each slide and multiplying by the slice thickness. The data is presented in the line graph. Data is shown as the mean ± standard error of the mean (SEM) **(D-F)**. No treatment: no treatment group; Long pep: long multi-epitope peptide; Long pep+Lena: long multi-epitope peptide plus lenalidomide; Lena+anti-PD1: Lenalidomide plus anti-PD1; Long pep+Lena+anti-PD1: long multi-epitope peptide plus lenalidomide and anti-PD1; Cocktail pep+Lena+anti-PD1: cocktail of multi-epitope peptide plus lenalidomide and anti-PD1. *P<* 0.05 (*).

The long multi-epitope peptide was synthesized using glycine linkers (represented by “G”) to link two tumor-associated antigen peptides (BIRC5^97-104^ and EphA2^682-689^) with a PADRE peptide, and a disulfide bond to create a dimeric fusion peptide (**Figure 1A** **)**. The treatment schedule is described in **Figure 1B**. The long multi-epitope peptide plus lenalidomide and anti-PD1 vaccine prolonged the overall survival of the mice (52.8 days ± 10.4 days) compared with the control group (24.1 days ± 1 day) (*p* = 0.046). However, there was no difference in survival between the control group, long multi-epitope peptide only (26.9 days ± 4.1 days), long multi-epitope peptide plus lenalidomide (24.2 days ± 1.2 days), lenalidomide plus anti-PD1 (28.4 days ± 5.8 days), and the cocktail of multi-epitope peptides plus lenalidomide and anti-PD1 (35.1 days ± 8.4 days) vaccines **(** **Figures 1C**, **S1**).

The long multi-epitope peptide plus lenalidomide and anti-PD1 vaccine also delayed tumor growth compared with the control group *(p =* 0.037) measured by magnetic resonance imaging (MRI). However, there was no difference in tumor growth between the control group, long multi-epitope peptides only, long multi-epitope peptide plus lenalidomide, lenalidomide plus anti-PD1, and the cocktail of multi-epitope peptides plus lenalidomide and anti-PD1 vaccines **(** **Figures 1D-F**, **S1** **)**. Further studies were done to highlight the immune-related therapeutic effects of the long multi-epitope peptide plus lenalidomide and anti-PD1 compared with the cocktail of multi-epitope peptides plus lenalidomide and anti-PD1 vaccines.

### The distribution of immune cells in the spleens, lymph nodes, and tumors

**Figure 2:**
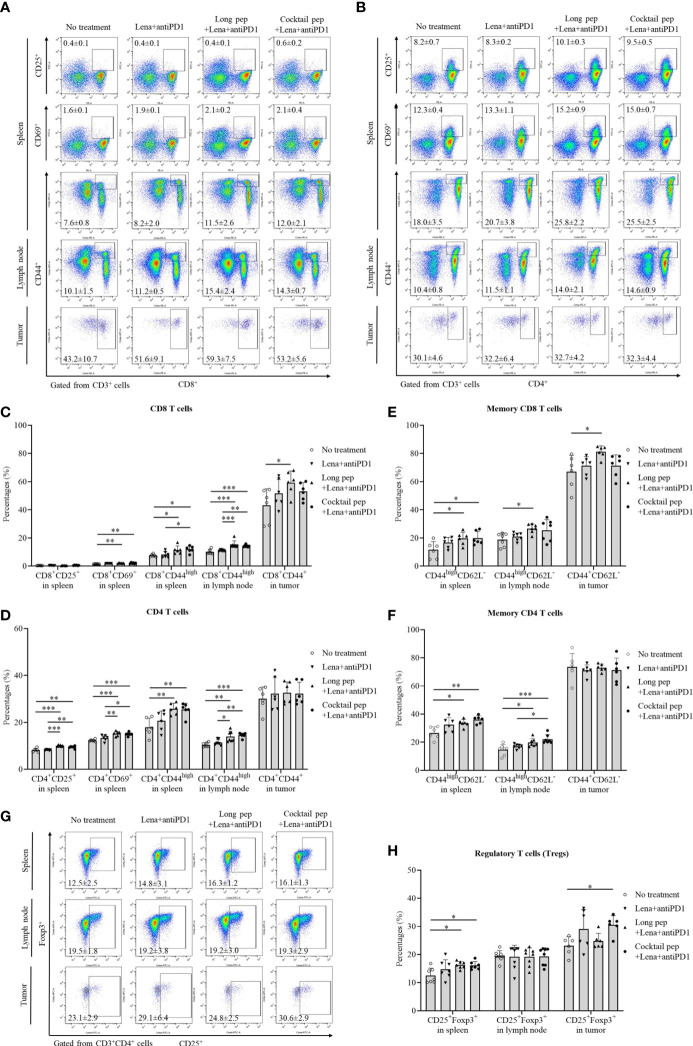
The distribution of immune cells in the spleens, lymph nodes, and tumors was confirmed by flow cytometry. The presentation of activated effector CD8 and CD4 T cells, such as activated CD8^+^CD25^+^, CD8^+^CD69^+^, and CD8^+^CD44^+^ or CD4^+^CD25^+^, CD4^+^CD69^+^, and CD4^+^CD44^+^ in spleens (n = 6 for each treatment group), lymph nodes (n = 7 for each treatment group), and tumors (n = 6 for each treatment group) were also measured **(A-D)**. Central memory and effector memory CD8^+^ and CD4^+^ T cells in spleens (CD44^high^CD62L^-^ and CD44^high^CD62L^+^) (n = 6 for each treatment group), lymph nodes (CD44^high^CD62L^-^ and CD44^high^CD62L^+^) (n = 7 for each treatment group), and tumors (CD44^+^CD62L^-^ and CD44^+^CD62L^+^) (n = 6 for each treatment group) were also measured **(E, F)**. The percentages of regulatory T cells (Tregs) (CD4^+^CD25^+^Foxp3^+^) in spleens (n = 7 for each treatment group), lymph nodes (n = 8 for each treatment group), and tumors (n = 6 for each treatment group) were determined **(G, H)**. All data is shown as the mean ± standard deviation (SD). No treatment: no treatment group; Long pep: long multi-epitope peptide; Long pep+Lena: long multi-epitope peptide plus lenalidomide; Lena+anti-PD1: Lenalidomide plus anti-PD1; Long pep+Lena+anti-PD1: long multi-epitope peptide plus lenalidomide and anti-PD1; Cocktail pep+Lena+anti-PD1: cocktail of multi-epitope peptide plus lenalidomide and anti-PD1. *p<* 0.05 (*), *p<* 0.001 (**), *p<* 0.0001 (***).

The difference in immune cell distributions in the spleens, lymph nodes, and tumors between lenalidomide plus anti-PD1, long multi-epitope peptide plus lenalidomide and anti-PD1, and the cocktail of multi-epitope peptides plus lenalidomide and anti-PD1 was analyzed using flow cytometry. The long multi-epitope peptide and the cocktail of multi-epitope peptides plus lenalidomide and anti-PD1 vaccines increased the percentage of activated CD8^+^ T cells and CD4^+^ T cells in the spleens and lymph nodes. However, only the long multi-epitope peptide plus lenalidomide and anti-PD1 vaccine increased the percentage of activated CD8^+^ T cells in the tumors (**Figures 2A-D** **)**. The percentages of CD8^+^CD69^+^ and CD8^+^CD44^high^ T cells in the spleens and CD8^+^CD44^high^ T cells in the lymph nodes were increased with the long multi-epitope peptide vaccine *(p =* 0.002, *p* = 0.027, and *p* = 0.000, respectively) and the cocktail of multi-epitope peptides plus lenalidomide and anti-PD1 vaccine (*p* = 0.002, *p* = 0.013, and *p* = 0.000, respectively) compared with the control group. Additionally, the percentages of CD4^+^CD25^+^, CD4^+^CD69^+^, and CD4^+^CD44^high^ T cells in the spleens, and CD4^+^CD44^high^ T cells in the lymph nodes were also enhanced with the long multi-epitope peptide plus lenalidomide and anti-PD1 vaccine *(p =* 0.000, *p* = 0.000, p = 0.003, and *p* = 0.001, respectively) and the cocktail of multi-epitope peptides plus lenalidomide and anti-PD1 vaccine (*p* = 0.001, *p* = 0.000, *p* = 0.005, and *p* = 0.000, respectively) compared with the control group. However, only CD8^+^CD44^+^ T cells were enhanced with the long multi-epitope peptide plus lenalidomide and anti-PD1 vaccine compared with the control group (*p* = 0.032).

The central and effector memory CD8^+^ and CD4^+^ T cells were also estimated in the spleens, lymph nodes, and tumors (**Figures 2E, F**, **S2** **)**. While the long multi-epitope peptide plus lenalidomide and anti-PD1 vaccine increased the percentage of effector memory CD8^+^ T cells compared with the control group in the spleens (CD44^high^CD62L^-^), lymph nodes (CD44^high^CD62L^-^), and tumors (CD44^+^CD62L^-^) (*p* = 0.041, *p* = 0.032, and *p* = 0.029, respectively). The cocktail of multi-epitope peptides plus lenalidomide and anti-PD1 vaccine only increased the percentage of effector memory CD8^+^ T cells in the spleens (*p* = 0.034). However, the long multi-epitope peptide and the cocktail of multi-epitope peptides plus lenalidomide and anti-PD1 vaccines increased the percentage of effector memory CD4^+^ T cells compared with the control group in the spleens (CD44^high^CD62L^-^) (*p* = 0.021 and *p* = 0.002, respectively) and the lymph nodes (CD44^high^CD62L^-^) (*p* = 0.016 and *p* = 0.000, respectively). There was no difference in the percentage of effector memory CD4^+^ T cells in the tumors (CD44^+^CD62L^-^). Although there was a difference in the percentage of effector memory T cells, there was no change in the percentage of central memory T cells or naïve T cells in spleens (CD44^high^CD62L^+^ or CD44^-^CD62L^+^), lymph nodes (CD44^high^CD62L^+^ or CD44^-^CD62L^+^), and tumors (CD44^+^CD62L^+^ or CD44^-^CD62L^+^) according to the treatments in this study.

The percentages of natural killer (NK) cells and regulatory T cells (Tregs) were also analyzed **(** **Figures S2**, **2G, H** **)**. Only the long multi-epitope peptide plus lenalidomide and anti-PD1vaccine showed increased percentages of NK cells in the lymph nodes compared with the control group *(p =* 0.018). While the cocktail of multi-epitope peptides plus lenalidomide and anti-PD1 vaccine increased the percentages of Tregs in both the spleens and tumors *(p =* 0.022 and *p* = 0.036, respectively), the long multi-epitope peptide combined lenalidomide and anti-PD1 vaccine only increased the percentages of Tregs in the spleens *(p =* 0.015). There was no difference in the percentages of Tregs in the lymph nodes.

### The expression of PD1 and PDL1 on immune cells and tumor

**Figure 3:**
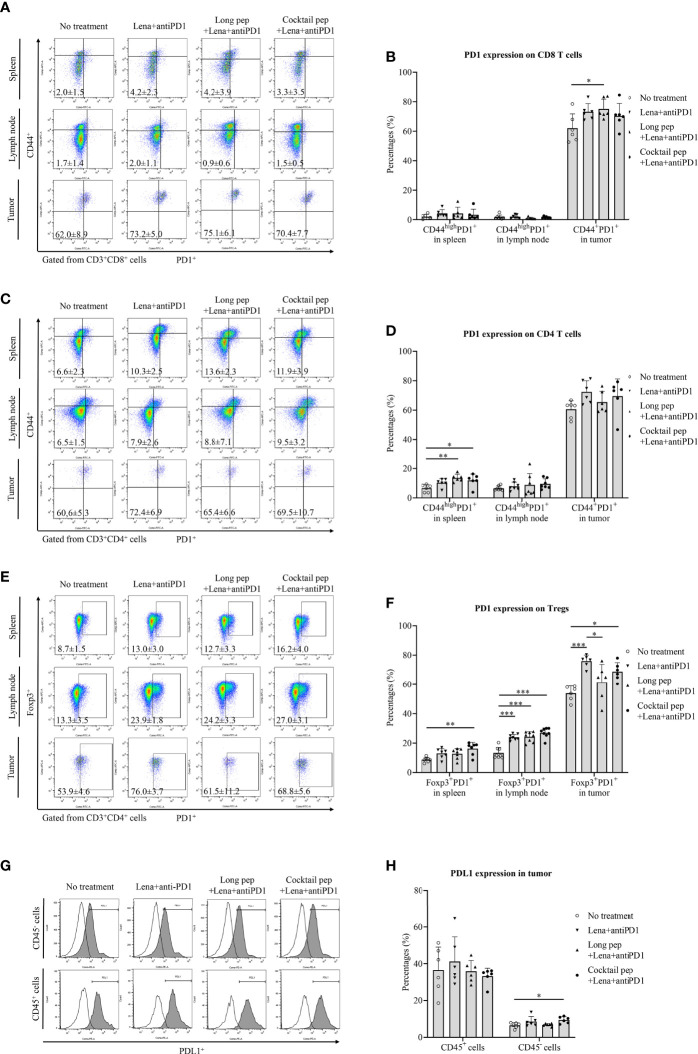
The expression of the inhibitory marker (PD1) and its ligand (PDL1) in immune cells and single tumor cells was confirmed by flow cytometry. The expression of PD1 in effector CD8^+^ and CD4^+^ T cells was measured in the spleens (n = 6 for each treatment group), lymph nodes (n = 7 for each treatment group), and tumors (n = 6 for each treatment group) **(A-D)**. The expression of PD1 in regulatory T cells (Tregs) in the spleens (n = 7 for each treatment group), lymph nodes (n = 8 for each treatment group), and tumors (n = 6 for each treatment group) was also measured **(E, F)**. The expression of PDL1 in immune and single tumor cells was measured (n = 6 for each treatment group) **(G, H)**. All data is shown as the mean ± standard deviation (SD). No treatment: no treatment group; Long pep: long multi-epitope peptide; Long pep+Lena: long multi-epitope peptide plus lenalidomide; Lena+anti-PD1: Lenalidomide plus anti-PD1; Long pep+Lena+anti-PD1: long multi-epitope peptide plus lenalidomide and anti-PD1; Cocktail pep+Lena+anti-PD1: cocktail of multi-epitope peptide plus lenalidomide and anti-PD1. *p<* 0.05 (*), *p<* 0.001 (**), *p<* 0.0001 (***).

The expression of PD1 on activated CD8^+^ T cells, CD4^+^ T cells, and Tregs in the spleens, lymph nodes, and tumors was estimated (**Figures 3A-F** **)**. While the long multi-epitope peptide plus lenalidomide and anti-PD1 vaccine primarily increased the expression of PD1 on activated CD8^+^ T cells (*p* = 0.039), the cocktail of multi-epitope peptides plus lenalidomide and anti-PD1 vaccine increased the expression of PD1 on Tregs (*p* = 0.014) in the tumors. While the cocktail of multi-epitope peptides plus lenalidomide and anti-PD1 vaccine increased the expression of PD1 on CD4^+^ T cells and Tregs (*p* = 0.036 and *p* = 0.002, respectively), the long multi-epitope peptide plus lenalidomide and anti-PD1 vaccine only increased the expression of PD1 on CD4^+^ T cells in the spleens (*p* = 0.005). In addition, the long multi-epitope peptide plus lenalidomide and anti-PD1 vaccine and the cocktail of multi-epitope peptides plus lenalidomide and anti-PD1 vaccine increased the expression of PD1 on Tregs in the lymph nodes (*p* = 0.000 and *p* = 0.000). There was no difference in PD1 expression on CD8^+^ and CD4^+^ T cells in the lymph nodes according to the different treatments. Additionally, while there was no difference in the expression of PDL1^+^ in CD45^+^ cells between the treatment groups, the expression of PDL1 was increased in the CD45^-^ cells from the cocktail of multi-epitope peptides plus lenalidomide and anti-PD1 vaccine group compared with the control group *(p =* 0.049) (**Figures 3G, H** **)**.

### Pro-inflammatory and anti-inflammatory cytokine production from re-stimulated splenocytes, re-stimulated lymph nodes, and single-tumor cells

**Figure 4:**
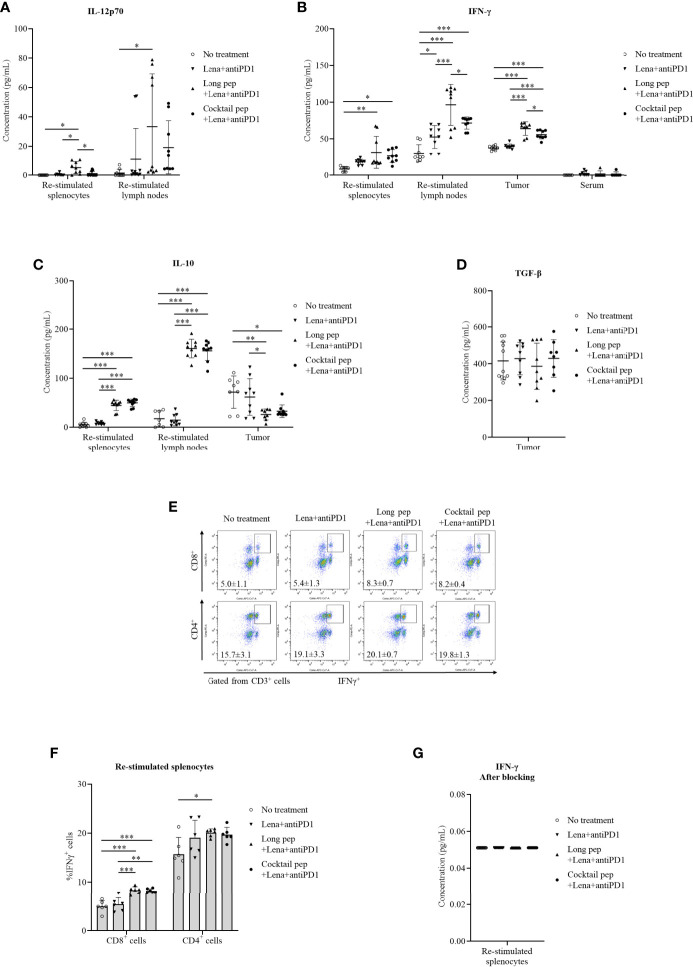
The pro-inflammatory and anti-inflammatory cytokine production in the culture media of re-stimulated splenocytes, re-stimulated lymph nodes, single tumor cells, and serum was determined *via* an ELISA. The released pro-inflammatory cytokines IL-12p70 were determined in the culture media of re-stimulated splenocytes and re-stimulated lymph nodes (No treatment: n = 9 and n = 9, Lena+anti-PD1: n = 9 and n = 7, long pep+Lena+anti-PD1: n = 9 and n = 9, and cocktail pep+Lena+anti-PD1: n = 7 and n = 9, respectively), IFN-γ were determined in the culture media of re-stimulated splenocytes, re-stimulated lymph nodes, single tumor cells, and serum (No treatment: n = 9, n = 9, n = 9, and n = 6, Lena+anti-PD1: n = 9, n = 9, n = 9, and n = 7, long pep+Lena+anti-PD1: n = 9, n = 9, n = 8, and n = 6, and cocktail pep+Lena+anti-PD1: n = 9, n = 9, n = 9, and n = 6, respectively) **(A, B)**. The anti-inflammatory cytokine (IL-10) levels were determined in the culture media of re-stimulated splenocytes, re-stimulated lymph nodes, and single tumor cells (No treatment: n = 10, n = 7 and n = 8, Lena+anti-PD1: n = 9, n = 9 and n = 9, long pep+Lena+anti-PD1: n = 9, n = 9 and n = 8, and cocktail pep+Lena+anti-PD1: n = 9, n = 9 and n = 10, respectively), TGF-β were determined in the culture media of single tumor cells (No treatment: n = 11, Lena+anti-PD1: n = 9, long pep+Lena+anti-PD1: n = 9, and cocktail pep+Lena+anti-PD1: n = 8, respectively), **(C, D)**. The percentages of CD8^+^IFN-γ^+^ cells and CD4^+^IFN-γ^+^ cells in the re-stimulated splenocytes and IFN-γ levels in the supernatant after membrane blocking for IFN-γ intracellular staining were calculated after 24 hours of re-stimulation (n = 6 mice per group) **(E-G)**. All data is shown as the mean ± standard deviation (SD). No treatment: no treatment group; Long pep: long multi-epitope peptide; Long pep+Lena: long multi-epitope peptide plus lenalidomide; Lena+anti-PD1: Lenalidomide plus anti-PD1; Long pep+Lena+anti-PD1: long multi-epitope peptide plus lenalidomide and anti-PD1; Cocktail pep+Lena+anti-PD1: cocktail of multi-epitope peptide plus lenalidomide and anti-PD1. *p<* 0.05 (*), *p<* 0.001 (**), *p<* 0.0001 (***).

The levels of pro-inflammatory cytokines, IL-12p70 and IFN-γ, and anti-inflammatory cytokines, IL-10 and TGF-β, were analyzed **(** **Figure 4** **)**. The levels of IL-12p70 were measured from the supernatant of re-stimulated splenocytes and lymph nodes. While the long multi-epitope peptide plus lenalidomide and anti-PD1 vaccine increased the production of IL-12p70 in the re-stimulated splenocytes and lymph nodes *(p =* 0.023 and *p* = 0.026, respectively), the cocktail of multi-epitope peptides plus lenalidomide and anti-PD1 vaccine group had stable IL-12p70 levels in the re-stimulated splenocytes and lymph nodes (**Figure 4A** **)**. In addition, both the long multi-epitope peptide plus lenalidomide and anti-PD1 vaccine and the cocktail of multi-epitope peptides plus lenalidomide and anti-PD1 vaccine increased the IFN-γ levels in the re-stimulated splenocytes (*p* = 0.002 and *p* = 0.015, respectively), re-stimulated lymph nodes (*p* = 0.000 and *p* = 0.000, respectively), and tumors (*p* = 0.000 and *p* = 0.000, respectively) compared with the control group. There was no difference in IFN-γ levels in the serum according to the treatment group. However, the long multi-epitope peptide plus lenalidomide and anti-PD1 vaccine increased the IFN-γ levels in the re-stimulated lymph nodes and tumors compared with the cocktail of multi-epitope peptides plus lenalidomide and anti-PD1 vaccine (*p* = 0.025 and *p* = 0.029, respectively) (**Figure 4B** **)**.

IL-10 levels were also measured in re-stimulated splenocytes, re-stimulated lymph nodes, and tumors (**Figure 4C** **)**. There was increased IL-10 expression in the re-stimulated splenocytes and lymph nodes with the long multi-epitope peptide plus lenalidomide and anti-PD1 vaccine *(p =* 0.000 and *p* = 0.000, respectively) and the cocktail of multi-epitope peptides plus lenalidomide and anti-PD1 vaccine *(p =* 0.000 and *p* = 0.000, respectively) compared with the control group. However, there were decreased IL-10 levels in the tumors with the long multi-epitope peptide and the cocktail of multi-epitope peptides plus lenalidomide and anti-PD1 compared with the control group (*p* = 0.008 and *p* = 0.018, respectively). Only the long multi-epitope peptide plus lenalidomide and anti-PD1 decreased IL-10 levels in tumors compared lenalidomide plus anti-PD1 group. There were no differences in the TGF-β levels in the tumors (**Figure 4D**).

The percentages of CD8^+^IFN-γ^+^ and CD4^+^IFN-γ^+^ T cells in the re-stimulated splenocytes were also analyzed (**Figures 4E-G** **)**. While the cocktail of multi-epitope peptides plus lenalidomide and anti-PD1 vaccine only increased the percentage of CD8^+^IFN-γ^+^ T cells in the spleens *(p =* 0.000), the long multi-epitope peptide plus lenalidomide and anti-PD1 vaccine increased the percentage of CD8^+^IFN-γ^+^ and CD4^+^IFN-γ^+^ T cells *(p =* 0.000 and *p* = 0.04, respectively) in the re-stimulated splenocytes after 24 hours of being stimulated with the peptides.

### The cytotoxic T lymphocytes (CTLs) function of re-stimulated splenocytes and lymph nodes

**Figure 5:**
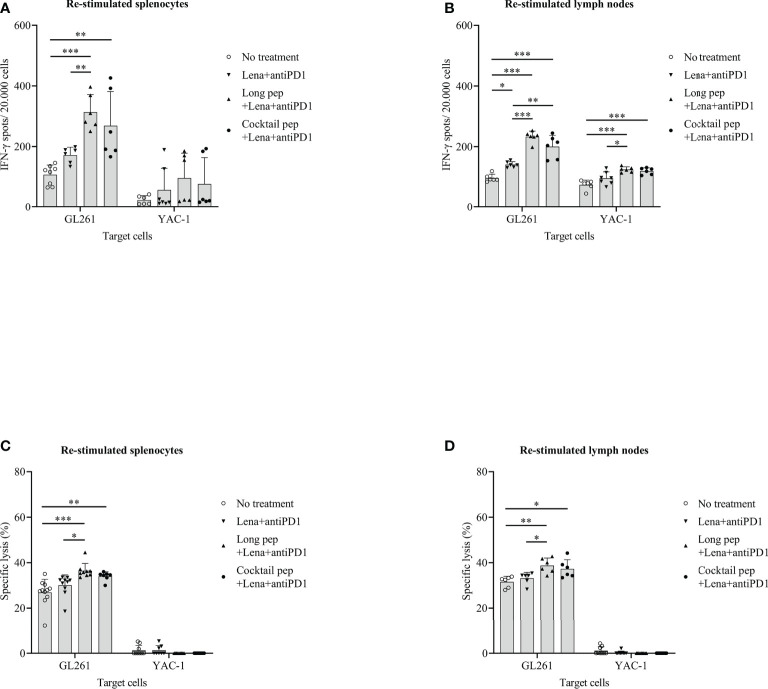
The cytotoxic T lymphocyte (CTL)-cell-mediated immune response of the re-stimulated splenocytes and lymph nodes from non-vaccinated and vaccinated mice were analyzed. IFN-γ secreted by re-stimulated splenocytes and lymph nodes when co-cultured with GL261 target cancer cells (No treatment: n = 8 and n = 6, Lena+anti-PD1: n = 6 and n = 6, long pep+Lena+anti-PD1: n = 6 and n = 6, and cocktail pep+Lena+anti-PD1: n = 6 and n = 6, respectively), and YAC-1 target cancer cells (No treatment: n = 6 and n = 6, Lena+anti-PD1: n = 7 and n = 7, long pep+Lena+anti-PD1: n = 6 and n = 6, and cocktail pep+Lena+anti-PD1: n = 6 and n = 6, respectively) was measured using the IFN-γ ELISPOT assay **(A, B)**. The specific killing effects of re-stimulated splenocytes and lymph nodes against GL261 target cancer cells (No treatment: n = 11 and n = 6, Lena+anti-PD1: n = 10 and n = 6, long pep+Lena+anti-PD1: n = 9 and n = 6, and cocktail pep+Lena+anti-PD1: n = 7 and n = 6, respectively), and YAC-1 target cancer cells (No treatment: n = 9 and n = 9, Lena+anti-PD1: n = 8 and n = 9, long pep+Lena+anti-PD1: n = 9 and n = 9, and cocktail pep+Lena+anti-PD1: n = 9 and n = 9, respectively) were measured using an LDH assay **(C, D)**. The GL261 and YAC-1 cell lines were used as specific and non-specific target cells for CTL sensitivity, respectively. All data is shown as the mean ± standard deviation (SD). No treatment: no treatment group; Long pep: long multi-epitope peptide; Long pep+Lena: long multi-epitope peptide plus lenalidomide; Lena+anti-PD1: Lenalidomide plus anti-PD1; Long pep+Lena+anti-PD1: long multi-epitope peptide plus lenalidomide and anti-PD1; Cocktail pep+Lena+anti-PD1: cocktail of multi-epitope peptide plus lenalidomide and anti-PD1. *p<* 0.05 (*), *p<* 0.001 (**), *p<* 0.0001 (***).

The CTLs-mediated immune responses of the re-stimulated splenocytes or lymph nodes from the non-vaccinated and vaccinated mice were measured. IFN-γ secretion by the re-stimulated splenocytes or lymph nodes after co-culturing with target cancer cells was investigated for the anti-tumor effects of the combined treatments against GL261 cells. Re-stimulated splenocytes or lymph nodes from the non-treated and treated mice were prepared for IFN-γ ELISPOT assays. The GL261 and YAC-1 cells were used as specific and non-specific target cells for investigating the CTL cell activity, respectively. In general, there were increased IFN-γ-secreting splenocytes and lymph nodes against GL261 cells with the long multi-epitope peptide plus lenalidomide and anti-PD1 vaccine *(p =* 0.000 and *p* = 0.000, respectively) and the cocktail of multi-epitope peptides plus lenalidomide and anti-PD1 vaccine (*p* = 0.001 and *p* = 0.000, respectively) compared with the control group. However, the long multi-epitope peptide plus lenalidomide and anti-PD1 vaccine showed a higher IFN-γ-secreting splenocytes and lymph nodes against GL261 cells compared with the control mice (*p* = 0.000 and *p* = 0.000, respectively), and the lenalidomide plus anti-PD1 vaccine (*p* = 0.005 and *p* = 0.000, respectively) **(** **Figures 5A, B** **)**.

In addition, the specific lysis of the re-stimulated splenocytes and re-stimulated lymph nodes against GL261 target cancer cells was confirmed. There was increased specific lysis in the re-stimulated splenocytes, and lymph nodes of mice given the long multi-epitope peptides plus lenalidomide and anti-PD1 vaccine *(p =* 0.000 and *p* = 0.004, respectively) and the cocktail of multi-epitope peptides plus lenalidomide and anti-PD1 vaccine (*p* = 0.009 and *p* = 0.025, respectively) compared with the control mice **(** **Figures 5C, D** **)**. Even though the long multi-epitope peptides plus lenalidomide and anti-PD1 vaccine showed little enhanced IFN-γ-secretion in splenocytes or lymph nodes, and the percentages of specific lysis were not significantly different in the long multi-epitope peptide plus lenalidomide and anti-PD1 and the cocktail of multi-epitope peptides plus lenalidomide and anti-PD1 treated mice. However, only the long multi-epitope peptide plus lenalidomide and anti-PD1 increased the specific lysis compared lenalidomide plus anti-PD1 group in re-stimulated splenocytes (*p* = 0.016), and re-stimulated lymph nodes (*p* = 0.029), respectively. In addition, the long multi-epitope peptides plus lenalidomide and anti-PD1 and the cocktail of multi-epitope peptides plus lenalidomide and anti-PD1 treated mice enhanced IFN-γ-secretion in lymph nodes against YAC-1 cells compared with the control group *(p =* 0.000 and *p* = 0.000, respectively). However, there was no difference in the specific lysis in the restimulated splenocytes and lymph nodes against YAC-1 cells.

## Discussion

Peptide cancer vaccines were developed based on targeting TSAs or TAAs as the immunotherapeutic epitopes to enhance immunogenicity and presentation to APCs (16, 17). Multi-peptide vaccines targeting various tumor antigens have been developed to overcome the limitations associated with single peptide vaccines, which are restricted to MHCI epitopes and target a single tumor antigen leading to immune escape of the tumor by loss of antigenicity (18–20). Among TAAs, BIRC5 and EphA2 are prognostic markers and therapeutic targets in various cancers, including GBM (16, 21–23). BIRC5 and EphA2 peptide vaccines have been shown to potentially affect GBM in preclinical and clinical trials (24–28). Moreover, the role of the PADRE in enhancing anti-tumor effects in peptide vaccines against GBM has been investigated in our previous study (29). Therefore, the anti-tumor effects of peptide vaccines targeting MHCI (BIRC5 and EphA2) and MHCII (PADRE) peptides were investigated using a vaccine composed of long multi-epitope peptides in this GL261 mouse model.

Although lenalidomide affects the proliferation of GBM cells, in previous studies, it failed to control tumor growth in the GBM mouse model (10, 30). However, it was found that lenalidomide induced proliferation and enhanced the persistent antitumor effect of CAR T cells *via* enhancing immunological synapses between the effector cells and the target cells (31). Multiple immunosuppression pathways coexist in the GBM microenvironment, which affects tumor progression and therapy outcomes (32, 33). Among them, immune checkpoints, such as the PD1/PDL1 axis, have renewed interest in immune-based cancer therapies due to their ability to prevent immunosuppression against tumors (34). In a previous study, anti-PD1 has shown potential effects in the treatment of GBM, and the combination of anti-PD1 with a peptide vaccine has shown beneficial results in GBM (35). Therefore, lenalidomide and anti-PD1 were used as adjuvants to modulate antitumor effects in peptide vaccines in this study. In this study, lenalidomide also reduced the proliferation of GL261 cells according to different treatment doses and interaction times *in vitro* (**Figure S3** **)**; however, the combination of lenalidomide and anti-PD1 did not reduce the tumor growth, and the role of lenalidomide in supporting immune cells stimulated with peptides was not observed in detail in this study.

The difference in immune responses against GBM in the mouse model related to the therapeutic effects between the cocktail of multi-epitope peptides vaccine and the long multi-epitope peptide in combination with lenalidomide and anti-PD1 vaccine was investigated in this study. While the long multi-epitope peptide plus lenalidomide and anti-PD1 vaccine prolonged mouse survival according to the reduced tumor volume, there was no difference in mouse survival with the cocktail of multi-epitope peptides plus lenalidomide and anti-PD1 vaccine. The therapeutic effects of the long multi-epitope peptide plus lenalidomide and anti-PD1 vaccine were paralleled with the dominant infiltration of immune cells and the release of cytokines by immune cells in the tumor. While Tregs inhibit antitumor immune responses, effector CTLs infiltrate the tumor site upon activation and take essential roles in killing cancer cells (36, 37). Although the function of re-stimulated splenocytes and lymph nodes against GL261 cells according to enhanced IFN-γ-secretion showed no difference between the long multi-epitope peptide vaccine and the cocktail of multi-epitope peptides plus lenalidomide and anti-PD1 vaccine, the long multi-epitope peptide plus lenalidomide and anti-PD1 vaccine demonstrated the increased function of re-stimulated splenocytes or lymph nodes against GL261 cells according to enhanced IFN-γ-secretion compared with control group and the lenalidomide plus anti-PD1 vaccine. Additionally, there was an increased infiltration of CD8 T cells and memory effector CD8 T cells with the long multi-epitope peptides plus lenalidomide and anti-PD1 vaccine and increased Treg infiltration with the cocktail of multi-epitope peptide with lenalidomide and anti-PD1 vaccine, which increased the antitumor response of the long multi-epitope peptide plus lenalidomide and anti-PD1vaccine in this study.

The released cytokines associated with the type of immune cells in the tumor were also analyzed in this study. While IL-10 is an anti-inflammatory cytokine-related to Treg function, IFN-γ is a pro-inflammatory cytokine related to CTL function (38, 39). The increased percentage of activated CD8^+^ T cells and Tregs cells in the tumors of the combination treatments paralleled the enhanced PD1 expression in these cells in this study. While the increased PD-1 expression on the tumor-infiltrating Tregs is related to their enhanced suppressive function (40), PD-1 expression on CTLs is a reflection of T cell activation, which inhibits T cell responses upon binding to PDL1. PD-1 expression on T cells in the context of cancer has been exclusively thought to be a marker of exhaustion (41, 42). In addition, PD1 expression was increased in activated CD8^+^ T cells with the long multi-epitope peptide plus lenalidomide and anti-PD1 vaccine, and increased PD1 expression in Tregs cells was observed with the cocktail of multi-epitope peptide plus lenalidomide and anti-PD1 vaccine in this study. Therefore, blocking the PD1 receptor by a PD1 antibody was expected to recover the dominant effects of CD8^+^ effector T cells with the long multi-epitope peptide combined lenalidomide vaccine and anti-PD1 and suppress Tregs with the cocktail of multi-epitope peptides plus lenalidomide and anti-PD1 vaccine. These actions lead to reduced IL-10 in tumors with the long multi-epitope peptide vaccine and the cocktail of multi-epitope peptides plus lenalidomide and anti-PD1 vaccine in tumors. However, only the long multi-epitope peptide plus lenalidomide and anti-PD1 decreased IL-10 levels compared lenalidomide plus anti-PD1 group in tumors. Moreover, the long multi-epitope peptide combined with lenalidomide and anti-PD1 vaccine illustrated dominant IFN-γ expression compared with the cocktail of multi-epitope peptides plus lenalidomide and anti-PD1 vaccine in the supernatant of single-tumor cells in this study. This study also showed that the long multi-epitope peptide plus lenalidomide and anti-PD1 is dominant in increased pro-inflammatory cytokine (IFN-γ) and decreased anti-inflammatory cytokine (IL-10) in tumors compared the cocktail of multi-epitope peptides plus lenalidomide and anti-PD1 group. The source of the enhanced IFN-γ was also clarified in this study, which primarily came from released CD8 T cells rather than CD4 T cells *via* detecting IFN-γ^+^CD8^+^ and IFN-γ^+^CD4^+^ T cells in splenocytes after re-stimulating with peptides.

Although the long multi-epitope peptide plus lenalidomide and anti-PD1 vaccine showed enhanced specific lysis against GBM target cells through enhanced IFN-γ secreting, the cells expressing inflammatory molecules, such as IFN-γ, IL-10, TNF-α, in the re-stimulated splenocytes and re-stimulated lymph nodes were not identified in this study. Although the function of each peptide, BIRC5^97-104^, EphA2^682-689^, and PADRE, in the long multi-epitope construct has not yet been evaluated, this study confirmed the potential to use glycine linkers (represented by “G”) to link MHCI peptides with MHCII peptides, and a disulfide bond to create a dimeric fusion peptide which forms the long multi-epitope peptides for cancer immunotherapy. This study also showed the potential to use the long multi-epitope construct composed of BIRC5^97-104^, EphA2^682-689^, and PADRE in combination with lenalidomide and anti-PD1 for the treatment of GBM. This treatment may be a useful treatment for other kinds of cancers that have high expressions of BIRC5 and EphA2. However, further studies should be done to clarify the function of each peptide in this combination in more detail. Additionally, this combination treatment was only confirmed in a single model; in future studies, several models should be investigated to highlight the effects of these combination treatments.

## Conclusion

This study shows the availability of using glycine linkers (represented by “G”) and a disulfide bond to create a dimeric fusion peptide for the long multi-epitope peptides construction in cancer treatment. Moreover, the long multi-epitope peptide constructed from MHCI (BIRC5 and EphA2) and MHCII (PADRE)-restricted peptides combined with adjuvants such as lenalidomide and anti-PD1 has the potential to enhance the immunotherapeutic effects in the GBM mouse model.

## Data availability statement

The original contributions presented in the study are included in the article/**Supplementary Material**. Further inquiries can be directed to the corresponding authors.

## Ethics statement

The animal study was reviewed and approved by Chonnam National University Animal Research Committee.

## Author contributions

T-A-TT, Y-HK, HY, T-YJ, and CL designed and performed the experiment. T-A-TT, Y-HK, G-EK, and T-YJ analyzed the data. T-A-TT and T-YJ wrote the article. SJ, I-YK, K-SM, Y-JK, T-KL, CL, H-JL, J-JL, and T-YJ contributed intellectually to the research. All authors contributed to the article and approved the submitted version.

## Funding

This study was supported by a grant (grant no. HCRI20004) from the Chonnam National University Hwasun Hospital Institute for Biomedical Science (Republic of Korea) and by the Basic Science Research Program through the National Research Foundation of Korea (NRF), funded by the Ministry of Science, ICT, and Future Planning (2020R1I1A3073338).

## Conflict of interest

The authors declare that the research was conducted in the absence of any commercial or financial relationships that could be construed as a potential conflict of interest.

## Publisher’s note

All claims expressed in this article are solely those of the authors and do not necessarily represent those of their affiliated organizations, or those of the publisher, the editors and the reviewers. Any product that may be evaluated in this article, or claim that may be made by its manufacturer, is not guaranteed or endorsed by the publisher.

## Supplementary material

Supplementary Figure 1 Kaplan-Meier survival curves were used to determine the survival of the mouse tumor model according to the different combination treatments in GL261 glioma-bearing mice (No treatment: n = 16, long pep: n = 9, long pep+Lena: n = 9, Lena+anti-PD1: n = 14, long pep+Lena+anti-PD1: n = 13, and cocktail pep+Lena+anti-PD1: n = 12) **(A)**. Brain tumor sizes after treatment with the different combinations were confirmed using magnetic resonance imaging (MRI) (No treatment: n = 8, long pep: n = 9, long pep+Lena: n = 8, Lena+anti-PD1: n = 9, long pep+Lena+anti-PD1: n = 10, and cocktail pep+Lena+anti-PD1: n = 9) **(B)**. No treatment: no treatment group; Long pep: long multi-epitope peptide; Long pep+Lena: long multi-epitope peptide plus lenalidomide; Lena+anti-PD1: Lenalidomide plus anti-PD1; Long pep+Lena+anti-PD1: long multi-epitope peptide plus lenalidomide and anti-PD1; Cocktail pep+Lena+anti-PD1: cocktail of multi-epitope peptide plus lenalidomide and anti-PD1. *p<* 0.05 (*), *p<* 0.001 (**), *p<* 0.0001 (***).

Supplementary Figure 2 Central memory and effector memory CD8^+^ and CD4^+^ T cells such as CD44^+^CD62L^-^ and CD44^+^CD62L^+^ on CD8^+^ and CD4^+^ T cells in spleens (n = 6 for each treatment group), lymph nodes (n = 7 for each treatment group), and tumors (n = 6 for each treatment group) were also measured **(A, B)**. Data was summarized by bar graph **(C-H)**. The percentages of natural killer (NK) cells (CD49b^+^CD69^+^ cells) in spleens and lymph nodes (n = 6 for each treatment group) were also defined **(I, J)**. All data is shown as the mean ± standard deviation (SD). No treatment: no treatment group; Long pep: long multi-epitope peptide; Long pep+Lena: long multi-epitope peptide plus lenalidomide; Lena+anti-PD1: Lenalidomide plus anti-PD1; Long pep+Lena+anti-PD1: long multi-epitope peptide plus lenalidomide and anti-PD1; Cocktail pep+Lena+anti-PD1: cocktail of multi-epitope peptide plus lenalidomide and anti-PD1. *p<* 0.05 (*), *p<* 0.001 (**), *p<* 0.0001 (***).

Supplementary Figure 3 The effects of lenalidomide on the proliferation of the GL261 cell line were measured by an MTT assay. The GL261 cells (5x10^3^ cells/well) were cultured with various doses of lenalidomide (2.5, 5, 10, and 20 µg/mL) for five days. The viability of GL261 cells was evaluated by using 3-(4,5-dimethylthiazol-2-yl)-2,5-diphenyltetrazolium bromide. The results showed a significant decrease in the viability of the GL261 cell line with increasing lenalidomide concentrations starting at day three. All data is shown as the mean ± standard deviation (SD). *p<* 0.05 (*), *p<* 0.001 (**), *p<* 0.0001 (***).
