# Supplementary figures and images for: The long multi-epitope peptide vaccine combined with adjuvants improved the therapeutic effects in a glioblastoma mouse model
Source: Front Immunol. 2022 Nov 9;13:1007285. doi: 10.3389/fimmu.2022.1007285 (PMC9681804; doi:10.3389/fimmu.2022.1007285)

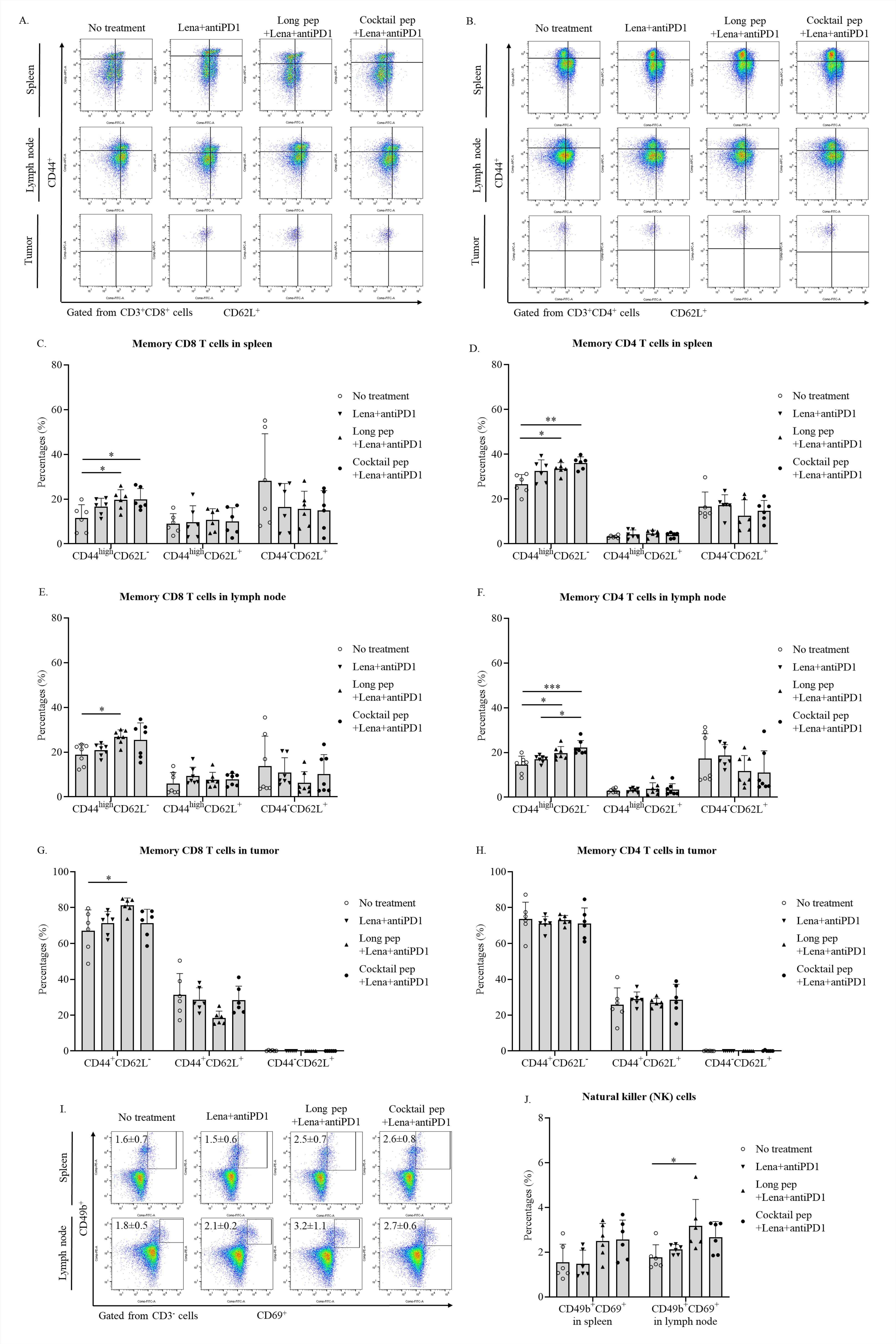

Supplement: Supplementary Figure 2 — Central memory and effector memory CD8+ and CD4+ T cells such as CD44+CD62L- and CD44+CD62L+ on CD8+ and CD4+ T cells in spleens (n = 6 for each treatment group), lymph nodes (n = 7 for each treatment group), and tumors (n = 6 for each treatment group) were also measured (A, B). Data was summarized by bar graph (C-H). The percentages of natural killer (NK) cells (CD49b+CD69+ cells) in spleens and lymph nodes (n = 6 for each treatment group) were also defined (I, J). All data is shown as the mean ± standard deviation (SD). No treatment: no treatment group; Long pep: long multi-epitope peptide; Long pep+Lena: long multi-epitope peptide plus lenalidomide; Lena+anti-PD1: Lenalidomide plus anti-PD1; Long pep+Lena+anti-PD1: long multi-epitope peptide plus lenalidomide and anti-PD1; Cocktail pep+Lena+anti-PD1: cocktail of multi-epitope peptide plus lenalidomide and anti-PD1. p< 0.05 (*), p< 0.001 (**), p< 0.0001 (***). [file Image_2.tiff]

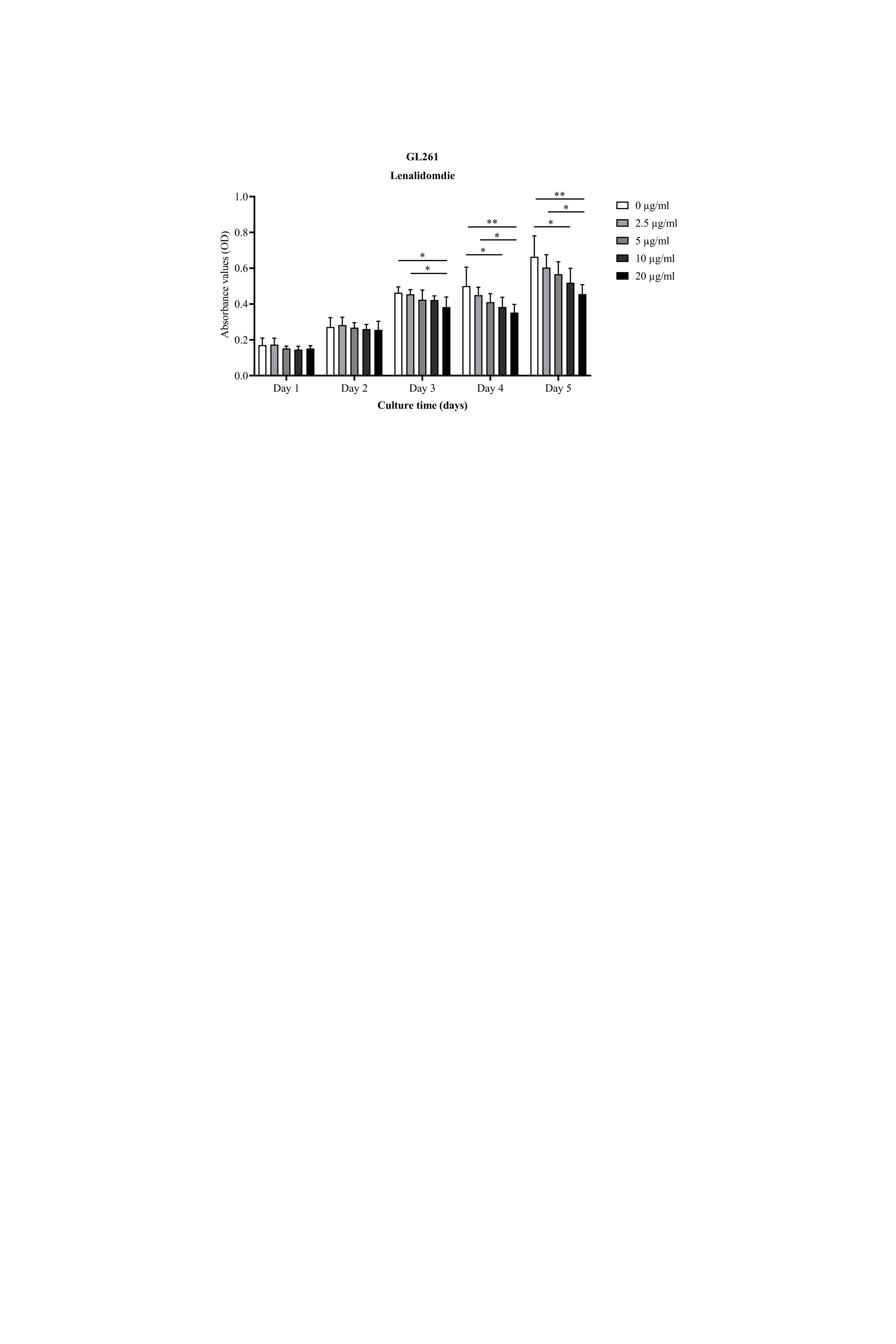

Supplement: Supplementary Figure 3 — The effects of lenalidomide on the proliferation of the GL261 cell line were measured by an MTT assay. The GL261 cells (5x10^3^ cells/well) were cultured with various doses of lenalidomide (2.5, 5, 10, and 20 µg/mL) for five days. The viability of GL261 cells was evaluated by using 3-(4,5-dimethylthiazol-2-yl)-2,5-diphenyltetrazolium bromide. The results showed a significant decrease in the viability of the GL261 cell line with increasing lenalidomide concentrations starting at day three. All data is shown as the mean ± standard deviation (SD). p< 0.05 (*), p< 0.001 (**), p< 0.0001 (***). [file Image_3.tif]
